# Determinants of choice of usual source of care among older people with cardiovascular diseases in China: evidence from the Study on Global Ageing and Adult Health

**DOI:** 10.1186/s12889-022-14352-w

**Published:** 2022-10-27

**Authors:** Tiange Xu, Katya Loban, Xiaolin Wei, Wenhua Wang

**Affiliations:** 1 School of Public Policy and Administration, Xi’an Jiaotong University, Xi’an, China; 2 Research Institute of the McGill University Health Centre, McGill University Health Centre, Montreal, Canada; 3 Dalla Lana School of Public Health, University of Toronto, Toronto, Canada

**Keywords:** Usual source of care, Cardiovascular diseases, Health care seeking, China

## Abstract

**Background:**

Cardiovascular diseases (CVD) are emerging as the leading contributor to death globally. The usual source of care (USC) has been proven to generate significant benefits for the elderly with CVD. Understanding the choice of USC would generate important knowledge to guide the ongoing primary care-based integrated health system building in China. This study aimed to analyze the individual-level determinants of USC choices among the Chinese elderly with CVD and to generate two exemplary patient profiles: one who is most likely to choose a public hospital as the USC, the other one who is most likely to choose a public primary care facility as the USC.

**Methods:**

This study was a secondary analysis using data from the World Health Organization’s Study on Global AGEing and Adult Health (SAGE) Wave 1 in China. 3,309 individuals aged 50 years old and over living with CVD were included in our final analysis. Multivariable logistic regression was built to analyze the determinants of USC choice. Nomogram was used to predict the probability of patients’ choice of USC.

**Results:**

Most of the elderly suffering from CVD had a preference for public hospitals as their USC compared with primary care facilities. The elderly with CVD aged 50 years old, being illiterate, residing in rural areas, within the poorest income quintile, having functional deficiencies in instrumental activities of daily living and suffering one chronic condition were found to be more likely to choose primary care facilities as their USC with the probability of 0.85. Among those choosing primary care facilities as their USC, older CVD patients with the following characteristics had the highest probability of choosing public primary care facilities as their USC, with the probability of 0.77: aged 95 years old, being married, residing in urban areas, being in the richest income quintile, being insured, having a high school or above level of education, and being able to manage activities living.

**Conclusions:**

Whilst public primary care facilities are the optimal USC for the elderly with CVD in China, most of them preferred to receive health care in public hospitals. This study suggests that the choice of USC for the elderly living with CVD was determined by different individual characteristics. It provides evidence regarding the choice of USC among older Chinese patients living with CVD.

**Supplementary Information:**

The online version contains supplementary material available at 10.1186/s12889-022-14352-w.

## Background

Cardiovascular diseases (CVD), the most common non-communicable diseases, are emerging as the leading contributor to death globally [1, 2]. The Global Burden of Disease Study (GBD) 2019 estimated that deaths caused by CVD reached approximately 18.56 million in 2019, with 24.70% occurring in China [3]. The prevalence of CVD in China increased from 4235.43 per 100,000 to 8460.08 per 100,000, and the incidence rate per 100,000 for CVD increased from 447. 81 to 867. 65 from 1990 to 2019 [3]. This increase induces an enormous economic burden of CVD, reaching over $2.87 trillion from 2010 to 2030, almost more than ten times that of South Korea [4]. Undoubtedly, there is an urgent need to improve the management of CVD.

Existing studies examining the choice of health care providers among patients living with CVD suggest that patients prefer to receive CVD-related health care in hospitals, particularly tertiary hospitals [5–7]. This preference for hospitals leads to unreasonable health care utilization and increasing medical expenditure. One study of stroke patients in China revealed that the average number of hemorrhagic stroke-related outpatient visits and hospital admissions per year in hospitals (mean of 0.71 outpatient visits and 0.11 hospital admissions) were higher than in primary care facilities (mean of 0.47 outpatient visits and 0.04 hospital admissions), with higher average medical cost per visit for outpatient (primary care facilities: $64.30, tertiary hospitals: $97.42) and inpatient visits (primary care facilities: $1,758.54, tertiary hospitals: $5,402.28) [8]. This suggests that patients’ preference for hospitals as their care provider is a key contributor to high economic burden of CVD.

However, there is an international consensus that community-based primary care is the optimal health care model for prevention and management of CVD. The American Heart Association Guide for Improving Cardiovascular Health at the Community Level gave recommendations for CVD prevention that can be implemented at the community level [9]. The 2016 European Guidelines on Cardiovascular Disease Prevention in Clinical Practice emphasized that CVD prevention and management should be delivered in primary care facilities, and that the general practitioner should be considered as the key professional to initiate and provide long-term health care for CVD patients [10]. The Korean government has also proposed to implement a community-based health care program for chronic diseases patients [11].

The health care delivery system in China has mainly included public hospitals and primary care facilities [12–14]. Of these medical institutions, public hospitals, with part of their revenues derived from government subsidies and health care fees, are owned by the government and could provide both specialist and primary health care services. Conversely, primary care facilities, mainly including community health centers in urban areas and township health centers and village clinics in rural areas, are responsible for delivering primary care and public health services and are a mixture of public and private ownership models [15, 16]. Individual service users report comparatively higher quality of health care, obtained at a higher price, in public hospitals than primary care facilities which were described as the health care system gatekeepers but had limited health care capacity, at a lower cost [12].

In China, people can choose any type of health care provider as their usual source of care (USC). USC is conceptualized as a regular place that a person visits most often for health care when needed, without restriction, and having a USC are associated with health care accessibility, the level of appropriate preventive care and treatment for chronic conditions, medical expenditure, and the prevalence of unmet health needs [17–28]. Some studies also pointed out that the effect of different types of USC on CVD management may vary. CVD patients using primary care facilities as a USC were more likely to experience good accessibility of care, have less emergency department visits and hospitalization, report higher awareness of their chronic conditions, and perceived stronger confidence in health management [26, 29]. While using the hospitals as a USC will result in negative outcomes of the above-mentioned aspects.

Understanding the determinants of USC choice and exploring which patients choose which types of health care provider as their USC will guide further health reform initiatives to better address the challenges of CVD. Previous studies have been conducted to examine the predictors of USC in other settings, such as diabetes care, acute upper respiratory tract infections care, older adults related care, with a focus on insurance, education, severity of illness, income, access to transportation, and so on [12, 30, 31]. However, there is insufficient evidence regarding the choice of USC among older patients living with CVD. In this study, we attempted to expand the existing research on the USC and address the knowledge gap. Based on the data collected by the World Health Organization (WHO) from eight provinces in China, we aimed to analyze the determinants of USC among the Chinese elderly with CVD, develop the nomograms, which are the graphical depictions of predictive statistical models and have been used for various clinical studies [32–34], to predict the probability of patients’ choice of USC, then generate the profiles of patients with the highest likelihood to choose primary care facilities or public hospitals as their USC. These findings will inform the current primary care based integrated health system reform in China.

## Methods

### Data source

The data were obtained from the WHO Study on Global AGEing and Adult Health (SAGE), which is a longitudinal study with nationally representative samples of individuals aged 50 + years old and one comparison sample of individuals aged 18–49 in six low- and middle-income countries [35]. Based on a multistage cluster sampling design, face-to-face interviews combined with standardized questionnaires were carried out, to collect information about socio-demographics, health risk factors and chronic conditions, health service utilization and patient responsiveness. SAGE Wave 1 2010 in China included 14,811 participants (13,175 individuals aged 50 years and above and 1,636 individuals aged 18–49) in eight provinces, with an overall response rate of 93% [35, 36].

### Study population

**Fig. 1:**
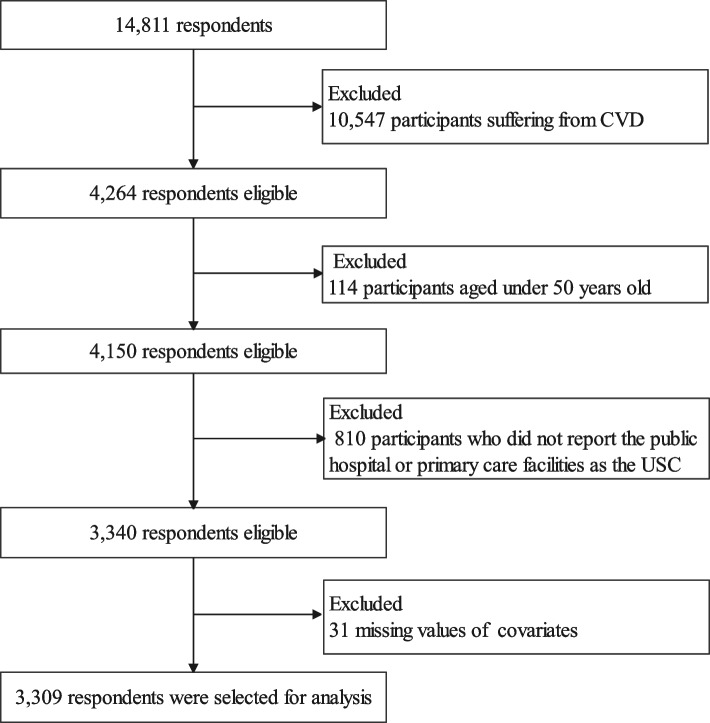
Flow chart for screening the analysis population

This study focused on the USC of the elderly with CVD. We selected the study population in the following steps in Fig. 1. Firstly, among the 14,811 respondents, 4,264 participants suffering from CVD (stroke, angina, and hypertension) were considered. Secondly, 114 participants aged under 50 years old were excluded and 4,150 participants remained. Thirdly, only participants who identified their USC as public hospitals or primary care facilities were selected. Thus, the data 810 participants who did not report the public hospital or primary care facilities as the USC were excluded. Fourth, 31 missing values in covariates (e.g., gender, age, and education) were excluded. The left 3,309 participants aged 50 years old and over with CVD and reported public hospitals or primary care facilities as their USC were included in our final analysis.

Respondents in the WHO SAGE-China were selected using a randomized sampling method [36]. First, 31 provinces were divided into eastern, central and western areas. Second, four provinces from eastern, two from the central and two from the western areas were selected. Thirdly, one county and one district were selected. In each country/district, four townships, two villages/enumeration areas per township/community, two residential blocks per village/enumeration area, and 42 households per residential block were chosen. Though the data is relatively old (2010), the choice of medical institutions among Chinese, particularly patients with chronic diseases, has not changed significantly in the past years [37–39]. Based on the above considerations, we maintain that our analysis could provide useful information for the whole patient population 50 years old and above living with CVD in China.

### Measurements

#### Usual source of care

The core dependent variable was the USC. In the SAGE survey, the USC was measured by one item: “Thinking about health care you needed in the last 3 years, where did you go most often when you felt sick or needed to consult someone about your health?” As mentioned above, only respondents who reported their USC as public hospitals or primary care facilities were eligible for inclusion. Both public clinics and private clinics were included in the primary care facilities group.

#### Control variables

Based on Andersen’s Behavioral Model, control variables for regression models were selected while considering previous relevant studies [40]. In this study, factors which can influence patients’ choice of USC can be divided into three categories.

1. Predisposing factors included gender, age, marriage, education, smoking, and alcohol consumption. Age was a continuous variable. Marriage was dichotomized into single versus current partnership. Education was grouped into four categories: illiterate, primary school, secondary school, and high school or above.
2. Enabling factors included residency, insurance and income quintile. The residency status included urban and rural. Insurance was a binary variable: yes or no. Income quintile was split into five groups: quintile 1 represented the poorest income group and quintile 5 represented the richest income group, which was based on a possession of a set of household assets and a number of dwelling characteristics [41–43].
3. Need factors included the health status, Body Mass Index (BMI), functional disability, depression, and chronic conditions. The health status was defined as three grades: good (comprising very good and good), moderate, and bad (comprising bad and very bad). The BMI was classified as four ranks: underweight, normal weight, overweight and obesity by the body mass index using the WHO criteria [44]. Activities of Daily Living (ADL) and Instrumental Activities of Daily Living (IADL) limitations were adopted to measure functional disability [45]. For the analysis, ADLs consisting of 16 items were classified as dichotomous variable according to whether respondents reported a limitation in one and above ADLs (Yes) and 0 (No) otherwise. IADLs were then dichotomized into a binary category: no deficiency consisting 1–3 limitations) and severe deficiency (consisting of 4–5 limitations). Depression (yes or no), derived form a set of 18 items, was used as a measurement of mental health [45]. Participants were asked if they had been diagnosed with any of the following chronic conditions: arthritis, angina, stroke, diabetes, chronic lung disease, asthma, depression, and hypertension. The number of common chronic conditions were divided into two categories: one, two and above [46].

### Data analysis

Descriptive statistics were used to examine the influence of factors on determinants of USC. Numbers and proportions were used to report participant characteristics. First, the chi-square and Kruskal–Wallis tests were conducted to examine the differences of participant characteristics among different types of USC. Second, two multivariable logistic regression models were employed to analyze determinants of USC. The first model was built to examine the determinants of public hospitals and primary care facilities. The second model was constructed to further analyze the determinants of public and private primary care facilities.

Then, based on multivariable logistic regression results, determinants were selected to formulate the nomogram (Nomogram A for the choice between primary care facilities and public hospitals, Nomogram B for the choice between public and private primary care facilities), which can be used to predict the probability of the choice of USC among the elderly with CVD. First, we calculated the score for each predictor variable (participant characteristics that were statistically significant in each regression model) based on their regression coefficient, then we added these scores. Second, the sum of all predictor variable scores was projected on the total points scale. Finally, the total point was transformed according to the probability of predicting USC. The discrimination of the nomogram was evaluated by calculating the concordance index (C-index), which ranged from 0.5 (no discrimination) to 1 (perfect discrimination). The calibration plot with 1,000 bootstrap resamples and Unreliability test were performed to assess the calibration. In this study, the nomograms had the C-index values of 0.76 (Nomogram A) and 0.73 (Nomogram B) and were well calibrated, which indicated that our nomograms were useful for assessing the choice of USC for the elderly with CVD.

Finally, sensitivity analyses were performed. Probit regression models were conducted to examine the association between the USC and influence factors. The results were consistent with our main findings.

Statistical significance was set at *P* < 0.05. All data analysis was conducted using STATA version 15.1.

## Results

### Participant characteristics

Of the 4,264 patients with CVD in the WHO SAGE-China, we identified 4,150 participants aged 50 years and above. 3,309 of these participants reported a USC and were therefore eligible for inclusion in our final analysis. Overall, 2,171 (65.61%) respondents reported public hospitals as their USC, and only 1,138 (34.39%) identified primary care facilities as their USC. Furthermore, primary care facilities were divided into private and public ownership, their respective proportions were 45.96% and 54.04%.

The characteristics of participants by type of USC are reported in Table 1. Compared with participants whose USC were public hospitals, participants who reported primary care facilities as their USC were more often female (55.32% of those who chose public hospitals vs 60.11% of those who chose primary care facilities), tended to be younger (66.58% vs 64.82%), were more educated (80.06% vs 65.99%), were more likely to live in rural areas (24.32% vs 62.92%) within the lowest income level (11.79% vs 26.45%), were more likely to report bad health status (28.01% vs 35.41%), and ADL limitations (70.15% vs 75.83%), were more likely to report functional deficiencies in IADLs (9.17% vs 13.36%), tended to suffer one chronic condition (38.92% vs 48.42%). Compared with individuals reporting private primary care facilities as their USC, individuals reporting public primary care facilities as their USC had higher mean age (64.03 yeas of those who chose private primary care facilities vs 65.49 yeas of those who chose public primary care facilities), were more likely to be married (77.44% vs 82.44%), to have a high school or above diploma (5.16% vs 13.82%) and medical insurance (79.92% vs 93.98%), to be urban residents (31.93% vs 41.46%), occupy the highest income quintile (5.16% vs 17.89%), report good health status (13.96% vs 20.49%) and without ADL limitations (18.93% vs 28.62%).

**Table 1:** Distribution of participant characteristics by different types of USC

Characteristics	Total (*n* = 3309)	USC		*P-*value	Total (*n* = 1138)	USC		*P-*value
		**Public hospitals** **(*n* = 2171)**	**Primary care facilities** **(*n* = 1138)**			**Private primary care facilities** **(*n* = 523)**	**Public primary care facilities** **(*n* = 615)**	
Gender, n (%)								
Male	1,424 (43.03)	970 (44.68)	454 (39.89)	0.008	454 (39.89)	197 (37.67)	257 (41.79)	0.157
Female	1,885 (56.97)	1,201 (55.32)	684(60.11)		684(60.11)	326 (62.33)	358 (58.21)	
Age, mean (SD)	65.98 (9.23)	66.58 (9.27)	64.82 (9.03)	< 0.001	64.82 (9.03)	64.03 (8.91)	65.49 (9.09)	0.006
Marriage, n (%)								
Single	623 (18.83)	397 (18.29)	226 (19.86)	0.272	226 (19.86)	118 (22.56)	108 (17.56)	0.035
Current partnership	2,686 (81.17)	1,774 (81.71)	912 (80.14)		912 (80.14)	405 (77.44)	507 (82.44)	
Education, n (%)								
Illiterate	820 (24.78)	433 (19.94)	387 (34.01)	< 0.001	387 (34.01)	208 (39.77)	179 (29.11)	< 0.001
Primary school	1,120 (33.85)	657 (30.26)	463 (40.69)		463 (40.69)	213 (40.73)	250 (40.65)	
Secondary school	656 (19.82)	480 (22.11)	176 (15.47)		176 (15.47)	75 (14.34)	101 (16.42)	
High school or above	713 (21.55)	601 (27.68)	112 (9.84)		112 (9.84)	27 (5.16)	85 (13.82)	
Residency, n (%)								
Urban	2,065 (62.41)	1,643 (75.68)	422 (37.08)	< 0.001	422 (37.08)	167 (31.93)	255 (41.46)	0.001
Rural	1,244 (37.59)	528 (24.32)	716 (62.92)		716 (62.92)	356 (68.07)	360 (58.54)	
Insurance, n (%)								
No	425 (12.84)	283 (13.04)	142 (12.48)	0.649	142 (12.48)	105 (20.08)	37 (6.02)	< 0.001
Yes	2,884 (87.16)	1,888 (86.96)	996 (87.52)		996 (87.52)	418 (79.92)	578 (93.98)	
Income quintile, n (%)								
Poorest	557 (16.83)	256 (11.79)	301 (26.45)	< 0.001	301 (26.45)	179 (34.23)	122 (19.84)	< 0.001
Q2	563 (17.01)	290 (13.36)	273 (23.99)		273 (23.99)	156 (29.83)	117 (19.84)	
Q3	671 (20.28)	455 (20.91)	217 (19.07)		217 (19.07)	85 (16.25)	132 (21.46)	
Q4	771 (23.30)	561 (25.84)	210 (18.45)		210 (18.45)	76 (14.53)	134 (21.79)	
Richest	747 (22.57)	610 (28.10)	137 (12.04)		137 (12.04)	27 (5.16)	110 (17.89)	
Health status, n (%)								
Bad	1,011 (30.55)	608 (28.01)	403 (35.41)	< 0.001	403 (35.41)	211 (40.34)	192 (31.22)	0.001
Moderate	1,634 (49.38)	1,098 (50.58)	536 (47.10)		536 (47.10)	239 (45.70)	297 (48.29)	
Good	664 (20.07)	465 (21.42)	199 (17.49)		199 (17.49)	73 (13.96)	126 (20.49)	
BMI, n (%)								
Underweight	75 (2.27)	45 (2.07)	30 (2.64)		30 (2.64)	15 (2.87)	15 (2.44)	0.111
Normal weight	1,654 (49.98)	1,089 (50.16)	565 (49.65)		565 (49.65)	277 (52.96)	290 (46.83)	
Overweight	1,154 (34.87)	753 (34.68)	401 (35.24)		401 (35.24)	176 (33.65)	225 (36.59)	
Obesity	426 (12.87)	284 (13.08)	142 (12.48)		142 (12.48)	55 (10.52)	89 (14.15)	
ADLs, n (%)								
No	923 (27.89)	648 (29.85)	275 (24.17)	0.001	275 (24.17)	99 (18.93)	176 (28.62)	< 0.001
Yes	2,386 (72.11)	1,523 (70.15)	863 (75.83)		863 (75.83)	424 (81.07)	439 (71.38)	
IADLs, n (%)								
No	2,958 (89.39)	1972 (90.83)	986 (86.64)	< 0.001	986 (86.64)	453 (86.62)	533 (86.67)	0.980
Yes	351 (10.61)	199 (9.17)	152 (13.36)		152 (13.36)	70 (13.38)	82 (13.33)	
Depression, n (%)								
No	3235 (97.76)	2130 (98.11)	1105 (97.10)	0.062	1105 (97.10)	505 (96.56)	600 (97.56)	0.315
Yes	74 (2.24)	41 (1.89)	33 (2.90)		33 (2.90)	18 (3.44)	15 (2.44)	
Chronic conditions, n (%)								
1	1,396 (42.19)	845 (38.92)	551 (48.42)	< 0.001	551 (48.42)	258 (49.33)	293 (47.64)	0.570
2 and above	1913 (57.81)	1,326 (61.08)	587 (51.58)		587 (51.58)	265 (50.67)	322 (52.36)	

*BMI* Body mass index, *ADLs* Activities of Daily Living, *IADLs* Instrumental Activities of Daily Living

### Determinants of patients’ choice of USC

#### Determinants of public hospitals and primary care facilities

The result of multivariable logistic regression analysis for USC is presented in Table 2. The differences between USC choices were statistically significant for age, education, residency, income quintile and chronic conditions. The probability of choosing primary care as USC decreased with increasing individuals’ age (*OR* = 0.974, *95% CI* = 0.964, 0.985). Rural residents (*OR* = 3.583, *95% CI* = 2.938, 4.370) were more inclined to report primary care facilities as their USC. Conversely, individuals who had a high school or above diploma (*OR* = 0.586, *95% CI* = 0.430, 0.798), higher income levels (Q3: *OR* = 0.563, *95% CI* = 0.434, 0.731; Q4: *OR* = 0.431, *95% CI* = 0.331, 0.561; Richest: *OR* = 0.333, *95% CI* = 0.249, 0.446), had IADL limitations (*OR* = 1.312, *95% CI* = 1.002,1.718), 2 and above chronic conditions (*OR* = 0.750, *95% CI* = 0.632, 0.890) were less willing to identify primary care facilities as their USC.

**Table 2:** Multivariable logistics regression of determinants associated with USC

Characteristics	Public hospitals and primary care facilities			Private and public primary care facilities		
	***OR***	***95%CI***	***P-*value**	***OR***	***95%CI***	***P-*value**
Gender (ref. = male)						
Female	1.006	0.845, 1.199	0.940	1.209	0.907, 1.110	0.196
Age	0.974	0.964, 0.985	< 0.001	1.051	1.033, 1.070	< 0.001
Marriage (ref. = single)						
Current partnership	0.881	0.706, 1.001	0.265	1.597	1.120, 2.276	0.010
Education (ref. = illiterate)						
Primary school	0.998	0.807, 1.234	0.983	1.448	1.054, 1.988	0.022
Secondary school	0.799	0.607, 1.054	0.112	1.583	1.011, 2.478	0.045
High school or above	0.586	0.430, 0.798	0.001	2.568	1.415, 4.659	0.002
Residency (ref. = urban)						
Rural	3.583	2.938, 4.370	< 0.001	0.697	0.40, 0.992	0.045
Insurance (ref. = no)						
Yes	0.844	0.656, 1.085	0.186	4.416	2.733, 7.136	< 0.001
Income quintile (ref. = poorest)						
Q2	0.891	0.690, 1.151	0.378	0.945	0.658, 1.357	0.759
Q3	0.563	0.434, 0.731	< 0.001	1.821	1.231, 2.694	0.003
Q4	0.431	0.331, 0.561	< 0.001	1.882	1.251, 2.831	0.002
Richest	0.333	0.249, 0.446	< 0.001	3.741	2.181, 6.420	< 0.001
Health status (ref. = bad)						
Moderate	1.187	0.976, 1.444	0.086	1.141	0.840, 1.551	0.399
Good	0.972	0.751, 1.259	0.832	1.479	0.973, 2.247	0.067
BMI (ref. = underweight)						
Normal weight	0.970	0.566, 1.663	0.912	0.990	0.433, 2.267	0.981
Overweight	1.111	0.644, 1.918	0.706	1.211	0.522, 2.811	0.655
Obesity	1.167	0.657, 2.073	0.598	1.571	0.644, 3.832	0.321
ADLs (ref. = no)						
Yes	1.045	0.854, 1.279	0.667	0.647	0.463, 0.905	0.011
IADLs (ref. = no)						
Yes	1.312	1.002,1.718	0.049	1.308	0.861,1.987	0.208
Depression (ref. = no)						
Yes	1.168	0.696,1.960	0.558	1.162	0.535, 2.522	0.705
Chronic conditions (ref. = 1)						
2 and above	0.750	0.632, 0.890	0.001	1.048	0.795, 1.382	0.738

*BMI* Body mass index, *ADLs* Activities of Daily Living, *IADL*s Instrumental Activities of Daily Living

#### Determinants of public and private primary care facilities

Table 2 also summarizes the results of a multivariable logistic regression model distinguishing between private and public primary care facilities as the USC. Our results demonstrate that there were significant differences in a number of factors, including age, marital status, education, residency, insurance status, income and health status. Participants were more likely to choose public primary care facilities as their USC with increasing age (*OR* = 1.051, *95% CI* = 1.033,1.070). Participants with higher educational attainment (primary school: *OR* = 1.448, *95% CI* = 1.054,1.988; secondary school: *OR* = 1.583, *95% CI* = 1.011,2.478; high school or above: *OR* = 2.568, *95% CI* = 1.415,4.659), who were married (OR = 1.597, *95% CI* = 1.120,2.276), who had medical insurance (*OR* = 4.416, *95% CI* = 2.733,7.136), who reported good economic conditions (Q3: *OR* = 1.821, *95% CI* = 1.231, 2.694; Q4: *OR* = 1.882, *95% CI* = 1.251, 2.831; Richest: *OR* = 3.741, *95% CI* = 2.181, 6.420) and reported without ADLs (*OR* = 0.647, *95% CI* = 0.463, 0.905) preferred to choose public primary care facilities as their USC.

### Sensitivity analyses

With respect to sensitivity analyses, we replaced multivariable logistics regression models with multivariable probit regression models to examine the determinants of USC. The results illustrate that age, education, residency, income quintile, IADLs and chronic conditions constituted the main factors influencing patients’ choice of primary care facilities. At the primary care level, the attributes that influenced the choice of USC were relatively similar to the determinants mentioned above and included age, marital status, educational attainment, residency, insurance status, income and ADLs (Table S1 in Supplementary File).

### The profiles of patients with highest likelihood of choosing primary care facilities as their USC

**Fig. 2:**
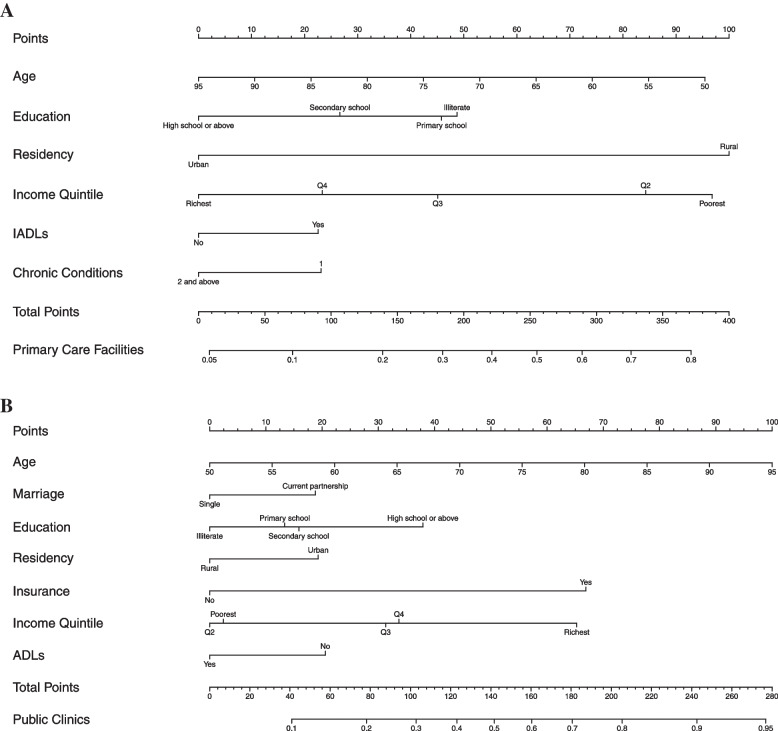
Nomogram for predicting the choice of USC among the elderly with CVD. **A** Nomogram for the choice between primary care facilities and public hospitals. **B** Nomogram for the choice between public and private primary care facilities. As an example, locate the “Age” and draw a line straight upward to the Points axis to determine the score of “Age”. Then repeat the process for each variable. Next, sum the scores for each covariate, and locate the sum on the Total Points axis. Finally, based on the sum scores, draw a straight line down to the probability axis (primary care facilities and public clinics axes) to determine the likelihood of primary care facilities or public clinics

The nomogram for predicting the choice between primary care facilities and public hospitals is shown in Fig. 2 (Nomogram A). The C-index of Nomogram A is 0.76 indicating a robust discrimination. The Unreliability test (*P* = 0.995 > 0.05) and calibration curve show a good agreement between prediction and observation in the probability of primary care facilities (Figure S1A in Supplementary File).

Older patients with CVD who had the highest likelihood of choosing primary care facilities as their USC, with the probability of 0.85, tended to have the following characteristics: aged 50 years old, being illiterate, living in rural areas, in the poorest income quintile, having IADLs and only having one chronic condition. Conversely, older patients with CVD aged 95 years old, who had a high school or above educational attainment, who resided in urban areas without IADL limitations, who were in the richest income quintile with 2 and above chronic conditions were least likely to choose primary care facilities as their USC, with the probability of 0.06 (Table S2 in Supplementary File).

### The profiles of patients with highest likelihood of choosing public primary care facilities as their USC

The nomogram for predicting the choice between public and private clinics is shown in Fig. 2 (Nomogram B). The nomogram shows a robust discrimination, with the C-index of 0.73. The Unreliability test (*P* = 0.856 > 0.05) and calibration curve indicates that the public clinics probabilities predicted by the nomogram agreed well with the actual probabilities (Figure S1B in Supplementary File).

Patients who were 95 years old, who were married, who lived within the richest income quintile, who were insured and resided in urban areas, who had a high school or above diploma and who did not report ADLs were more likely to choose public primary care facilities as their USC, with the probability of 0.77. Conversely, the lowest probability of identifying public primary care facilities as the USC was 0.09, which often occurred in patients with CVD aged 50 years old, who were single and illiterate, without insurance, residing in rural areas, within Q2 income quintile, and who reported had ADLs. These patients were more likely to report private primary care facilities as their USC (Table S3 in Supplementary File).

## Discussion

This study analyzed the determinants of USC among 3,309 Chinese people aged 50 and over living with CVD. We found that most of the elderly suffering from CVD had a preference for public hospitals as their USC compared with primary care facilities, and that the determinants of choice of USC varied according to USC type: age, education, residency, income quintile, IADl limitations, and chronic conditions were the major influencing factor in determining the choice between public hospitals and primary care facilities; while age, marital status, education, residency, insurance status, income and ADLs played decisive role for the choice between private and public primary care facilities. Our study contributes to the knowledge of USC and its determinants among older people with CVD in China.

The older CVD patients with the following characteristics tended to have the highest possibility of reporting primary care facilities as their USC with the probability of 0.85: aged 50 years old, being illiterate, living in rural areas, within the poorest income quintile, reporting functional deficiencies in IADLs and having one chronic condition. Conversely, the elderly with CVD aged 95 years old, had high school or above education level, resided in urban areas without IADL limitations, were in richest income quintile with 2 and above chronic conditions had the highest probability of choosing public hospitals as their USC.

One of the key determinants of the choice of USC between public hospitals and primary care facilities was age: the likelihood of reporting public hospitals as their USC increased significantly with age. This finding is similar to the results reported in a prior study in Nigeria [47]. Aging was characterized by the deterioration of physical function, which resulted in the increased severity of the disease and a demand for a higher capacity for diagnosis and treatment [47, 48]. Other studies reported that as the progressive complexity of diseases increased with age, patients were more likely to experience comorbidity, thereby, were more likely to need a higher quality of health care [49–54]. We also found that older CVD patients with comorbidity preferred to choose public hospitals as their USC. In China, despite the recent improvements in primary care, primary care facilities still struggle to attract highly qualified professionals and suffer from insufficient supplies of medicines necessary for chronic communicable disease management, which may give rise to more unmet health needs of the elderly with CVD [55, 56]. Conversely, given the comparatively higher capacity (including the availability of highly qualified health care professionals, medical equipment and technology), accurate diagnoses and appropriate treatments in the case of complex patients, the health care needs of the elderly with CVD can be adequately addressed in public hospitals [12, 46]. However, our finding differed from the results of some previous research suggesting that as patients got older they were expected to prefer primary care facilities for chronic disease management [57, 58]. This difference may be attributed to the differences in primary care capabilities in different regions. The differences between study participants may also lead to this inconsistency.

Educational attainment was another significant factor affecting older patients’ choice of USC. Older patients with higher educational attainment were more inclined to report public hospitals instead of primary care facilities as their USC. This finding is consistent with previous research in some low- and middle- income countries [47, 59]. A prior study of 1,549 patients with hypertension living in China suggested that well-educated older patients were more likely to choose hospitals for health care [60]. There was strong evidence indicating that individuals with higher educational levels tended to have better health literacy, higher socioeconomic status, and therefore were more likely to exercise choice for and access superior health care in hospitals [61–65].

From the perspective of residency, we found that older patients residing in rural areas had a significantly higher probability of choosing primary care facilities as their USC, confirming the findings of previous studies from China [66–68]. This finding may be partly explained by the fact that rural residents tend to be poorer than those settled in urban areas, hence they cannot afford health care at a higher price from hospitals [69, 70]. One study conducted in Shanghai confirmed that the elderly with chronic diseases who had limited financial means were more likely to choose community health facilities over hospitals [5]. Furthermore, the distance between medical institutions and patient’s place of residence may affect the choice of USC [69, 71]. In comparison with hospitals, primary care facilities are predominantly located in rural areas, such as villages or townships, and are easily accessible to rural patients.

Income was another important determinant in patients’ USC preference, which is consistent with the findings of a study conducted in three rural areas in China [71]. Our study has shown that individuals in the Q2 quintile and above were significantly more likely to identify public hospitals as their USC than those in the poorest income quintile. One possible explanation is that patients in higher income groups had higher purchasing power, and thus can afford the long-term burden of medical expenditures incurred in public hospitals [72, 73]. Moreover, the preference for higher quality of health care services may also influence their choice [47, 70].

In terms of IADLs, we found that IADLs increased the likelihood of choosing primary care facilities as the USC of elderly with CVD. Functional deficiencies in IADLs usually leads to a failure in the ability to carry out basic functional activities, especially for older adults [74]. CVD elderly had difficulties with getting where you want to go, transporting themselves to places where they want to go, and using public transport, as well as the long distance between public hospitals and their residence, thus, they were more likely to regarding primary care facilities as the USC [69, 71, 75].

With respect to primary care facilities’ determinants among the elderly suffering from CVD, a 95 years old citizen who was married, within the richest income quintile, who was insured, who had a high school or above diploma and did not report ADLs had the highest probability of choosing a public primary care facility as his or her USC, with the probability of 0.77.

We found that a patient’s sociodemographic characteristics, particularly age, marital status and educational attainment, were significant influencing factors impacting their choice of USC. Age has proven to be the strongest predictor of USC choice between private and public primary care facilities. This result suggests that the likelihood of choosing public primary care facilities as a USC increased with age. As discussed previously, owing to the poor health with increased age, patients were more likely to prefer chronic disease management in public primary care facilities in which they perceived a high quality of health care [47, 48]. A national study conducted in China indicated that public primary care facilities performed better in health care quality than private primary care facilities in relation to the following domains: prompt attention, communication and autonomy, dignity and confidentiality [46]. In contrast, patients in India preferred private health care providers over public health care providers [72, 76]. However, the health care delivery system in India is different from China. For example, private health care in China is still underdeveloped, suffering from a shortage of highly qualified medical professionals and lower service efficiency [77].

We have also observed that patients living with their spouses preferred to identify public primary care facilities as USC. Previous research has shown that marital status may be related to financial security and social connection, therefore married patients with CVD may have more resources to afford medical costs in public health care facilities [78, 79]. In addition, owing to the high education levels of patients, they tended to have better health literacy, higher income status and stable social support, would like to regard public primary care facilities as the USC for high quality of health care [61–65].

Apart from those factors, our study further demonstrated that patients with insurance were more likely to identify public primary care facilities as their USC. Public medical institutions are included in the medical insurance benefit packages, meaning that patients can get reimbursement when they have medical consultations in public primary care facilities, which can relieve the economic burden to a certain extent. In contrast, patients visiting private primary care medical institutions for health care have to cover medical expenditures by themselves. Besides, income was another factor influencing the choice of USC. Individuals with relatively high-income levels and residing in urban arears have been shown to be inclined to identify public primary care facilities as USC. Patients who earned higher-income and living in urban settles tended to have better capacity for payment on health care, price of health care services could not limit their health care seeking behavior and their pursuit of high-quality health care, so they have more opportunities to access the public primary care facilities and regard them as the USC [47, 69, 70, 72, 73].

Finally, factors like ADLs also had an important impact on the preference of USC between private and public primary care facilities. Patients without ADLs were more willing to choose public primary care facilities as their USC. This may be related to their higher health literacy and emphasis given to health status, which may influence the decision to seek more standardized and high-quality health care from public health care facilities. Previous research demonstrated that public primary care facilities can provide a more reliable supply of medicines, while private primary care facilities tended to be poorly regulated and suffered from low quality of prescribing [80]. Moreover, the elderly patients with ADLs tended to identify the private primary care facilities as their USC, which could be explained by the higher health care costs of long-term ADLs care need and the cheaper care provided by private primary care facilities [80–83].

### Limitations

Our study had several limitations. First, the SAGE data did not cover all CVDs. Further studies analyzing the determinants of USC in patients living with other types of CVD are needed. Second, this study used data from a cross-sectional survey in 2010 because the second round of survey data is still in the process of collation and is still not available. Thus, this study conducted a cross-sectional analysis using the first round of survey data, which could not infer causality between participant characteristics and types of USC. However, due to the rigorous design of the SAGE study, accurate and reliable data collection, and good representativeness of samples, it can reflect the overall situation of Chinese elderly patients with CVDs. In addition, we performed sensitivity analyses, and the results were consistent with the findings of our main analysis, which can explain the determinants of USC to some extent. Future work could leverage longitudinal research to examine causality in our findings. Third, we could not clearly separate public hospitals into different ranks. Further research could analyze the determinants according to different ranks of public hospitals. Fourth, due to the limitation of the questionnaire, the present study was subject to possibly unobserved confounding factors, such as cognitive function, living arrangement, number of surviving adult children, and so on. Future studies may indeed benefit from examining the impact of these characteristics on the choice of usual source of care among the elderly suffering from CVD. Despite the limitations, our findings add to the knowledge of which factors may influence USC choices among the Chinese elderly with CVD and generate two exemplary patient profiles: one who is most likely to choose a public hospital as the USC, the other one who is most likely to choose a public primary care facility as the USC, with the probability of their choice of USC.

## Conclusions

Our findings suggest that whilst public primary care facilities are the optimal USC for the elderly with CVD in China, but most of them preferred to gain health care in public hospitals. We also found that the choice of USC in older patients living with CVD in China was determined by the variety of individual characteristics. Patients with CVD who were aged 95 years old, being married, within the richest income quintile, residing in urban areas, being insured, having a high school or above diploma and reporting ADLs were most likely to choose public primary care facilities as their USC. This study provides evidence regarding the choice of USC among older Chinese patients living with CVD. Efforts to further improve chronic disease management should be continued to cope with various levers, such as improving the quality of chronic disease management in primary care, exploring effective guidelines for the comorbidity management. increasing the proportion of reimbursement in primary care facilities, implementing long-term care insurance, enhancing patients’ health literacy by health education.

## Supplementary Information

**Additional file 1:** **Table S1.** Multivariable probit regression of determinants associated with USC. **Fig. S1.** The calibration curves for the nomogram. **Table S2.** Probability of the elderly with CVD who would most or be least likely to choose primary care facilities as their USC. **Table S3.** Probability of the elderly with CVD who would most or be least likely to choose public primary care facilities as their USC.

## Data Availability

Information on the details of data is equally available via the webpage https://apps.who.int/healthinfo/systems/surveydata/index.php/catalog/sage. The data that support this study is available from the WHO Study on SAGE, but restrictions apply to the availability of these data, which were used under license for the current study.

## Abbreviations

USC: Usual source of care
CVD: Cardiovascular diseases
WHO: World Health Organization
SAGE: Study on Global AGEing and Adult Health
GBD: Global Burden of Disease Study
ADL: Activities of Daily Living
IADL: Instrumental Activities of Daily Living
BMI: Body Mass Index
