# Supplementary material for: Determinants of choice of usual source of care among older people with cardiovascular diseases in China: evidence from the Study on Global Ageing and Adult Health
Source: BMC Public Health. 2022 Oct 27;22:1970. doi: 10.1186/s12889-022-14352-w (PMC9615328; doi:10.1186/s12889-022-14352-w)
Supplement: Supplementary file 1 — Additional file 1: Table S1. Multivariable probit regression of determinants associated with USC. Fig. S1. The calibration curves for the nomogram. Table S2. Probability of the elderly with CVD who would most or be least likely to choose primary care facilities as their USC. Table S3. Probability of the elderly with CVD who would most or be least likely to choose public primary care facilities as their USC. [file 12889_2022_14352_MOESM1_ESM.docx]

**Supplementary File**

Table S1 Multivariable probit regression of determinants associated with USC

Fig. S1 The calibration curves for the nomogram

Table S2 Probability of the elderly with CVD who would most or be least likely to choose primary care facilities as their USC

Table S3 Probability of the elderly with CVD who would most or be least likely to choose public primary care facilities as their USC

**Table S1** Multivariable probit regression of determinants associated with USC

| **Characteristics** | **Public hospitals and primary care facilities** | | |  | **Private and public primary care facilities** | | |
| --- | --- | --- | --- | --- | --- | --- | --- |
|  | ***β*** | ***95%CI*** | ***P-*value** |  | ***β*** | ***95%CI*** | ***P-*value** |
| Gender (ref.= male) |  |  |  |  |  |  |  |
| Female | 0.002 | -0.101, 0.106 | 0.962 |  | 0.120 | -0.054, 0.294 | 0.176 |
| Age | -0.015 | -0.022, -0.009 | <0.001 |  | 0.030 | 0.019, 0.040 | <0.001 |
| Marriage (ref.= single) |  |  |  |  |  |  |  |
| Current partnership | -0.073 | -0.204, 0.059 | 0.280 |  | 0.286 | 0.073, 0.498 | 0.009 |
| Education (ref.= illiterate) |  |  |  |  |  |  |  |
| Primary school | -0.005 | -0.133, 0.123 | 0.842 |  | 0.226 | 0.033, 0.420 | 0.022 |
| Secondary school | -0.141 | -0.306, 0.023 | 0.092 |  | 0.270 | 0.001, 0.540 | 0.049 |
| High school or above | -0.305 | -0.483, -0.127 | 0.001 |  | 0.568 | 0.214, 0.922 | 0.002 |
| Residency (ref.= urban) |  |  |  |  |  |  |  |
| Rural | 0.775 | 0.655, 0.894 | <0.001 |  | -0.218 | -0.428, -0.005 | 0.045 |
| Insurance (ref.= no) |  |  |  |  |  |  |  |
| Yes | -0.088 | -0.238, 0.062 | 0.250 |  | 0.892 | 0.610, 1.175 | <0.001 |
| Income quintile (ref.= poorest) |  |  |  |  |  |  |  |
| Q2 | -0.075 | -0.230, 0.081 | 0.346 |  | -0.028 | -0.249, 0.193 | 0.805 |
| Q3 | -0.354 | -0.511, -0.197 | <0.001 |  | 0.366 | 0.128, 0.605 | 0.003 |
| Q4 | -0.512 | -0.670, -0.354 | <0.001 |  | 0.393 | 0.145, 0.641 | 0.002 |
| Richest | -0.649 | -0.820, -0.478 | <0.001 |  | 0.802 | 0.487, 1.117 | <0.001 |
| Health status (ref.= bad) |  |  |  |  |  |  |  |
| Moderate | 0.102 | -0.015, 0.218 | 0.087 |  | 0.081 | -0.104, 0.266 | 0.391 |
| Good | -0.021 | -0.174, 0.132 | 0.789 |  | 0.244 | -0.009, 0.496 | 0.058 |

**Table S1 (continued)**

| **Characteristics** | **Public hospitals and primary care facilities** | | |  | **Private and public primary care facilities** | | |
| --- | --- | --- | --- | --- | --- | --- | --- |
|  | ***β*** | ***95%CI*** | ***P-*value** |  | ***β*** | ***95%CI*** | ***P-*value** |
| BMI (ref.= underweight) |  |  |  |  |  |  |  |
| Normal weight | -0.034 | -0.351, 0.283 | 0.833 |  | -0.009 | -0.489, 0.507 | 0.972 |
| Overweight | 0.044 | -0.276, 0.365 | 0.782 |  | 0.123 | -0.382, 0.629 | 0.633 |
| Obesity | 0.076 | -0.262, 0.414 | 0.660 |  | 0.293 | -0.244, 0.830 | 0.285 |
| ADLs (ref.= no) |  |  |  |  |  |  |  |
| Yes | 0.023 | -0.096, 0.141 | 0.709 |  | -0.261 | -0.462, -0.059 | 0.011 |
| IADLs (ref.= no) |  |  |  |  |  |  |  |
| Yes | 0.156 | -0.006, 0.328 | 0.060 |  | 0.162 | -0.094, 0.418 | 0.215 |
| Depression (ref.= no) |  |  |  |  |  |  |  |
| Yes | 0.088 | -0.225, 0.340 | 0.583 |  | 0.118 | -0.346, 0.582 | 0.618 |
| Chronic conditions (ref.= 1) |  |  |  |  |  |  |  |
| Yes | -0.166 | -.268, -0.064 | 0.001 |  | 0.031 | -0.137, 0.98 | 0.720 |

BMI = body mass index. ADLs= Activities of Daily Living. IADLs= Instrumental Activities of Daily Living

**A**


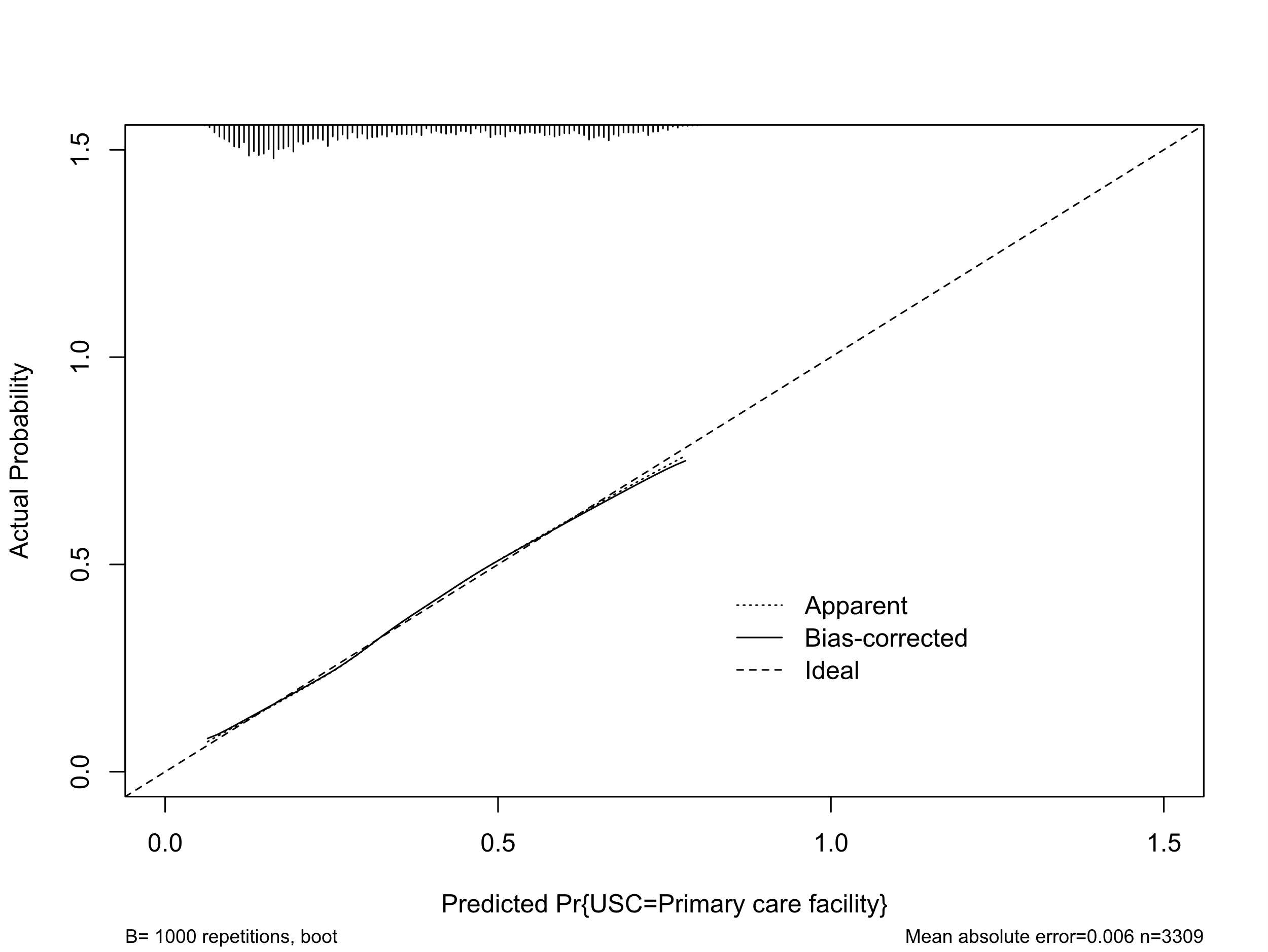


**B**


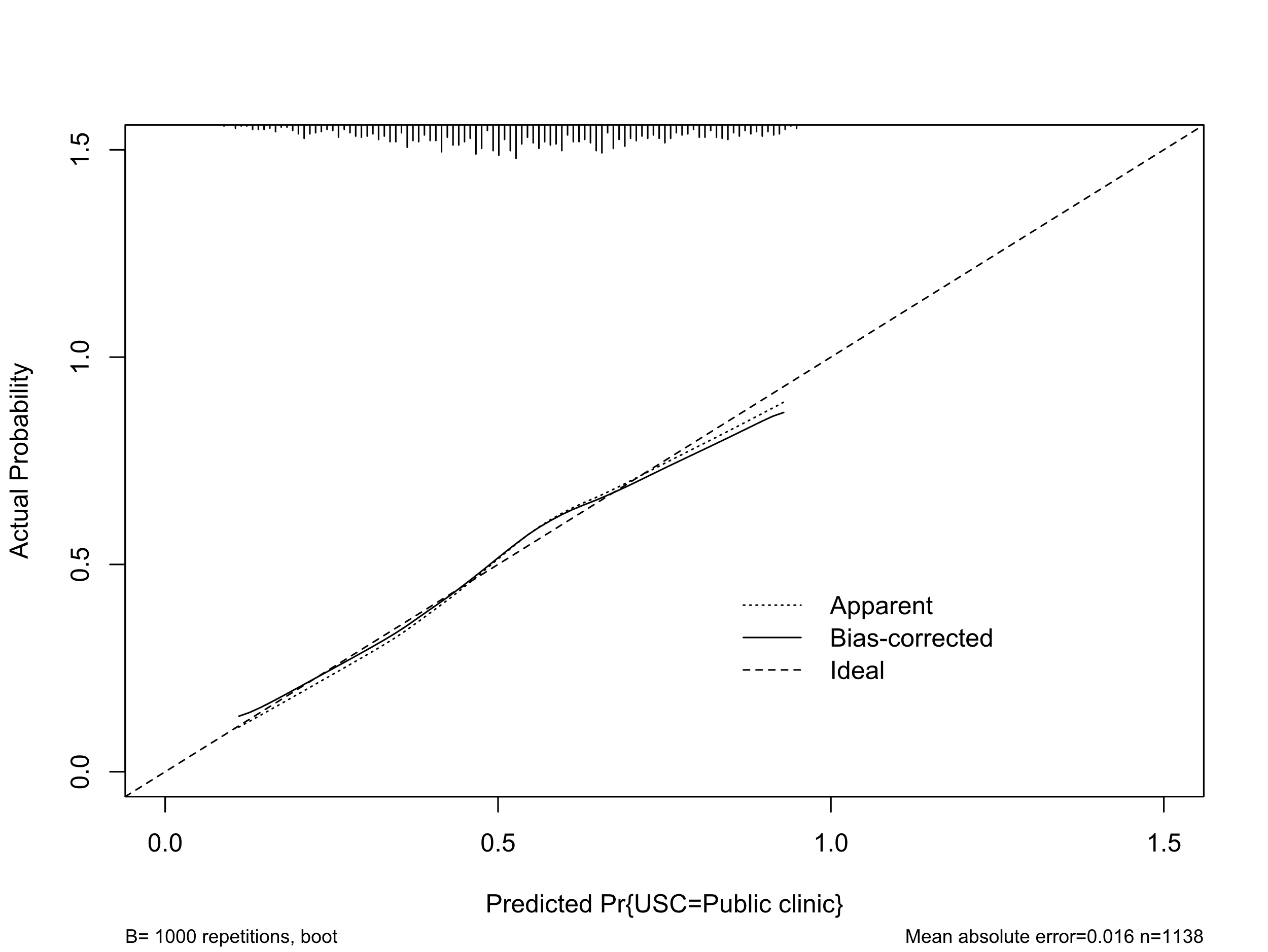


**Fig. S1** The calibration curves for the nomogram. A for Nomogram A. B for Nomogram B.

**Table S2** Probability of the elderly with CVD who would most or be least likely to choose primary care facilities as their USC

| **Variables** | **Patients who would be most likely to choose primary care facilities as USC** | | | |  | **Patients who would be least likely to choose primary care facilities as USC** | | | |
| --- | --- | --- | --- | --- | --- | --- | --- | --- | --- |
|  | **Variable value** | **Point** | **Total points** | **Probability** |  | **Variable value** | **Point** | **Total points** | **Probability** |
| Age | 50 years old | 95.43 | 386.62 | 0.85 |  | 95 years old | 0 | 0 | 0.06 |
| Education | Illiterate | 48.73 | - | - |  | High school or above | 0 | - | - |
| Residency | Rural | 100 | - | - |  | Urban | 0 | - | - |
| Income quintile | Poorest | 96.81 | - | - |  | Richest | 0 | - | - |
| IADLs | Yes | 22.55 | - | - |  | No | 0 | - | - |
| Chronic conditions | 1 | 23.12 | - | - |  | 2 and above | 0 | - | - |

Patients who would be least likely to choose primary care facilities as USC means patients who would be most likely to choose public hospitals as USC.

IADLs= Instrumental Activities of Daily Living.

**Table S3** Probability of the elderly with CVD who would most or be least likely to choose public primary care facilities as their USC

| **Variables** | **Patients who would be most likely to choose public primary care facilities as USC** | | | |  | **Patients who would be least likely to choose public primary care facilities as USC** | | | |
| --- | --- | --- | --- | --- | --- | --- | --- | --- | --- |
|  | **Variable value** | **Point** | **Total points** | **Probability** |  | **Variable value** | **Point** | **Total points** | **Probability** |
| Age | 95 years old | 100 | 328.73 | 0.77 |  | 50 years old | 0 | 0 | 0.09 |
| Marriage | Current partnership | 18.80 | - | - |  | Single | 0 | - | - |
| Education | High school or above | 37.91 | - | - |  | Illiterate | 0 | - | - |
| Residency | Urban | 19.30 | - | - |  | Rural | 0 | - | - |
| Insurance | Yes | 66.89 | - | - |  | No | 0 | - | - |
| Income quintile | Richest | 65.28 | - | - |  | Q2 | 0 | - | - |
| ADLs | No | 20.56 | - | - |  | Yes | 0 | - | - |

Patients who would be least likely to choose public primary care facilities as USC means patients who would be most likely to choose private primary care facilities as USC.

ADLs= Activities of Daily Living.
